# Neurotropic EV71 causes encephalitis by engaging intracellular TLR9 to elicit neurotoxic IL12-p40-iNOS signaling

**DOI:** 10.1038/s41419-022-04771-3

**Published:** 2022-04-11

**Authors:** Rai-Hua Lai, Yen-Hung Chow, Nai-Hsiang Chung, Tsan-Chi Chen, Feng-Shiun Shie, Jyh-Lyh Juang

**Affiliations:** 1 Institute of Molecular and Genomic Medicine, National Health Research Institutes, Zhunan, Taiwan, ROC; 2 National Institute of Infectious Disease and Vaccinology, National Health Research Institutes, Zhunan, Taiwan, ROC; 3 Graduate Institute of Biomedical Sciences, China Medical University, Taichung, Taiwan, ROC; 4 Institute of Molecular and Cellular Biology, National Tsing Hua University, Hsinchu, Taiwan, ROC; 5 Center for Neuropsychiatric Research, National Health Research Institutes, Zhunan, Taiwan, ROC

**Keywords:** Toll-like receptors, Viral infection

## Abstract

Brainstem encephalitis, a manifestation of severe enterovirus 71 (EV71) infection, is an acute excessive inflammatory response. The mechanisms underlying its development remain poorly understood. Usually neurotropic viruses trigger acute host immune response by engaging cell surface or intracellular receptors. Here, we show that EV71 engagement with intracellular receptor TLR9 elicits IL-12p40-iNOS signaling causing encephalitis in mice. We identified IL-12p40 to be the only prominent cytokine-induced at the early infection stage in the brainstem of mice subjected to a lethal dose of EV71. The upregulated IL-12p40 proteins were expressed in glial cells but not neuronal cells. To better understand the role of IL-12p40 in severe EV71 infection, we treated the EV71-infected mice with an antibody against IL-12p40 and found the mortality rate, brainstem inflammation, and gliosis to be markedly reduced, suggesting that the acute IL-12p40 response plays a critical role in the pathogenesis of brainstem encephalitis. Mechanistically, intracellular TLR9 was found essential to the activation of the IL-12p40 response. Blocking TLR9 signaling with CpG-ODN antagonist ameliorated IL-12p40 response, brainstem inflammation, and limb paralysis in mice with EV71-induced encephalitis. We further found the glial IL-12p40 response might damage neurons by inducing excess production of neurotoxic NO by iNOS. Overall, EV71 engagement with intracellular TLR9 was found to elicit a neurotoxic glial response via IL12p40-iNOS signaling contributing to the neurological manifestation of EV71 infection. This pathway could potentially be targeted for the treatment of brainstem encephalitis.

## Introduction

Enterovirus 71 (EV71), the most neurovirulent of over 100 serotypes of enterovirus species, usually leads to hand-foot-and-mouth disease (HFMD) in young children in the Asia-Pacific region [1, 2]. Over the past decade, enteroviruses have been estimated to have infected over 13 million people worldwide causing more than three thousand deaths [3, 4]. Brainstem encephalitis is reported to be the most serious neurological manifestation of enterovirus infections, usually progressing to fatal neurogenic pulmonary edema and cardiopulmonary failure [5]. How EV71 causes brainstem encephalitis is poorly understood, and thus this disease is currently untreatable.

EV71-induced encephalitis is an acute, exaggerated immune defense response in which the immune system mistakenly attacks the brainstem. Because the brainstem controls both breathing and heart rate, its dysfunction can lead to neurogenic pulmonary edema [6–9]. Of the various inflammatory cytokines, endogenous IL-12 is unique in that it is essential for early control of pathogenic infections. IL-12 p40 mRNA expression was first reported about two decades ago to be transiently increased shortly after infection with several different viruses in mice [10]. Around the same time, the early induction of IL-12 was reported to activate innate immune responses and differentiation of Th1 CD4+ cells upon infection [11]. It is interesting to note that, although IL-12 can protect against infection, IL-12 deficient mice have been found to have less virus-induced encephalitis, a finding that suggests that IL-12 signaling might play a role in the development of viral encephalitis [12], though the role of its signaling has not been studied in the context of EV71-induced encephalitis.

Most molecular studies of EV71 infection have focused on its effect on systemic cytokines and systemic immune responses, not local immune response. In this study, we investigate whether EV71 infection causes brainstem encephalitis via dysregulation of local immune response. To do this, we performed a series of animal studies to explore inflammatory patterns in the brainstem using mouse-adapted EV71 virus (MP4) and hSCARB2-Tg mouse models to identify the specific cytokine(s) produced locally in the brainstem. These two models were also used to characterize the role that these cytokines might play in the development of brainstem encephalitis.

## Materials and methods

### Mice

The 7-day-old ICR mice were inoculated oral infection or intraperitoneal injection with MP4 virus, or medium alone as described by Chun-Keung Yu [13]. The mice were monitored daily for pathological signs and were sacrificed at various time points following inoculation. Similarly, the young hSCARB2-Tg or non-TG wildtype mice at 7-day of age were inoculated subcutaneous injection with EV71 5746 strains, or medium alone. The investigators were not blinded to the experimental design and data analysis. The sample sizes for each experiment were determined according to the “Sample Size Determination” from “Guidelines for care and uses of mammals in neuroscience and behavioral research” [14]. Mice were allocated randomly in all experiments. Viral VP1 expression and CNS syndromes were verified in all the animals with or without viral injection. The severity of CNS syndromes was scored from 0 to 5 using the following criteria; 5 = severe front and rear limb paralysis (LP) and no movement, 4 = moderate 2 rear LP and hesitant movement, 3 = one rear LP with bending legs, 2 = mild rear limb bended, 1 = slightly rear limb bended, 0 = normal movement. LP is defined as the rigidness of mouse legs that are hesitant to move. All experimental animal procedures and protocols were approved by the Institutional Animal Care and Use Committee at NHRI (approved protocol no. NHRI-IACUC-099007-A).

### Dissection of brainstem and cerebrum

The mice were sacrificed at the indicated times post-inoculation, and the muscle attachments to the skulls were immediately removed, the scalp was cut along the midline of the skull, and the cranial skeleton was removed. The skull was then inverted to allow brain to fall from the skull to assist in removal of the brain. The dissected brain was placed on a prechilled stainless-steel block and cleaved into two cerebral hemispheres along the longitudinal fissure. The brainstem (including midbrain, pons, and medulla) and cerebellum were dissected out and immediately transferred to a labeled vial and stored at −80 °C for further RNA extraction or protein immunoassay. For paraffin-embedded tissues, the tissues were immediately fixed in 4% paraformaldehyde.

### Primary cultures, cell culture, viruses, and cell viability assay

Primary microglia and neurons cultured cells were derived from cortices of postnatal day 1 to day 3 of hSCARB2-Tg mice; the primary cultures were performed as described previously [15, 16]. The following cell lines including SH-SY5Y (human neuroblastoma, ATCC CRL-2266), U87-MG (human glioma, ATCC CCL-127) were cultured in MEM (Invitrogen, USA). Cells were grown at 37 °C in a 5% CO_2_ humid atmosphere and checked for mycoplasma contamination. The following enterovirus strains including mouse-adapted EV71 strain MP4 (GenBank: GQ150746.1), which was obtained from Dr. Chun-Keung Yu (National Cheng Kung University, Tainan), Tainan/5746/98 (C2) (GenBank: AF304457.1), TW/2272/98 (GenBank Accession no. AF119795), and 5079 of CVA16 (GenBank: AF177911.1) were propagated in Vero or RD cells based on the microcarrier cell culture bioprocess. The virus stocks were stored at −80 °C until use. Cell viability was evaluated with the formazan-based WST-1 assay (Roche), according to the manufacturer’s instructions. The absorbance of samples was measured at 450 nm against a reference at 690 nm with a spectrophotometer.

### Antibody and recombinant protein

The antibodies used in this study are listed as follows: anti-IL-12p40 (H306), Santa Cruz Biotechnology, Cat# SC-7926; GFAP, abcam, AB53554; GFAP (GA5), Cell Signaling Technology, Cat# 3670; anti-TLR9, Abcam, ab134368; anti-PARP (H250), Santa Cruz Biotechnology, Cat# SC-7150; anti-GAPDH, GeneTex, Cat# GTX100118. Primary antibodies used for neutralization include p40 (C17.8), or isotype control antibodies, BD Biosciences. The recombinant proteins used in this study to treat SHS-Y5Y cell lines: Murine IL-12 p70; Murine IL-12 p40 and Murine IL-12 p80, PeproTech.

### Cytokine antibody microarray and IL-12p40 ELISA assays

The total cell lysate from the brainstem or cerebrum was analyzed using the mouse Cytokine panel C1 (RayBiotech) according to the manufacturer’s instructions. The kit can detect 22 common cytokines simultaneously in a single sample (detail panel shown in Fig. S1C). An IL-12p40 ELISA assay (Elabscience Biotechnology) was used to detect serum IL-12p40 levels in the EV71-infected hSCARB2-Tg mice.

### Quantitative real-time reverse transcription-PCR

Total RNAs from brainstem tissues or culture cells were extracted using the illustra™ RNAspin Mini RNA Isolation Kit (GE Healthcare Life Sciences) for reverse transcription with the High-Capacity cDNA Reverse Transcription Kits (ABI Applied Biosystems, USA) according to the manufacturer’s instructions. The quantitative real-time reverse transcription-PCR (qPCR) analysis was performed using the Fast SYBR Green Master Mix (ABI Applied Biosystems, USA). The gene expression levels were calculated by the relative standard curve method. The primers used for qPCR in this study are listed as follows: IL-12p35: 5ʹ-ACGAGAGTTGCCTGGCTACTAG-3ʹ and 5ʹ-CCTCATAGATGCTACCAAGGCAC-3ʹ; IL-12p40: 5ʹ-CAGAAGCTAACCATCTCCTGGTTTG-3ʹ and 5ʹ-TCCGGAGTAATTTGGTGCTTCACAC-3ʹ; IL-23p19: 5ʹ-GCCCCGTATCCAGTGTGAAG-3ʹ and 5ʹ-CGGATCCTTTGCAAGCAGAA-3ʹ; IFNγ: 5ʹ-GGGTTGTTGACCTCA AACTTGGCA-3ʹ and 5ʹ-CAGGCCATCAGCAACAACAT-3ʹ; TNFα: 5ʹ-CTACTCCCAGGTTCTCTTCAA-3ʹ and 5ʹ-GCAGAGAGGAGGTTGACTTTC-3ʹ; IL6: 5ʹ-TACCACTTCACAAGTCGGAGGC-3ʹ and 5ʹ-CTGCAAGTGCATCATCGTTGTTC-3ʹ; TLR9: 5ʹ-TTCTCAAGACGGTGGATCGC-3ʹ and 5ʹ-GCAGAGGGTTGCTTCTCACG-3ʹ; TLR3: 5ʹ-GTCTTCTGCACGAACCTGACAG and 5ʹ-TGGAGGTTCTCCAGTTGGACCC-3ʹ; TLR7: 5ʹ-GTGATGCTGTGTGGTTTGTCTGG-3ʹ and 5ʹ-CCTTTGTGTGCTCCTGGACCTA-3ʹ; TLR8: 5ʹ-AAGTGCTGGACCTGAGCCACAA-3ʹ and 5ʹ-CCTCTGTGAGGGTGTAAATGCC-3ʹ; iNOS: 5ʹ-GAGACAGGGAAGTCTGAAGCAC-3ʹ and 5ʹ-CCAGCAGTAGTTGCTCCTCTTC-3ʹ; pan-enterovirus-specific primer: 5ʹ-GTGTGAAGAGTCTATTGAGC-3ʹ and 5ʹ-ATTGTCACCATAAGCAGCCA-3ʹ; GAPDH: 5ʹ-CATCACTGCCACCCAGAAGACTG-3ʹ and 5ʹ-ATGCCAGTGAGCTTCCCGTTCAG-3ʹ.

### Immunostaining assay

Immunostaining assays are described previously [17]. Briefly, the collected brainstem tissues were immediately fixed in 4% paraformaldehyde for 8–12 h and later embedded in paraffin wax. The paraffin sections were deparaffinized, rehydrated, and followed by antigen retrieval. The sections were then incubated in blocking reagent and then treated with a specific antibody overnight at 4 °C. The following day the sections were incubated with fluorescence-conjugated secondary antibody for 1 h at room temperature and examined with a fluorescent microscope.

### Quantification and statistical analysis

Sample sizes (number of patients or mice), mean, and statistical significance values are indicated in the Figure legends. Experiments were performed at least in triplicate. All the groups were statistically compared. Statistical significance was determined using Graphpad Prism 6 and p values are indicated by stars, *p < 0.05, **p < 0.01 and ***p < 0.001.

## Results

### EV71 infection induces an acute IL-12p40 response in the brainstem

**Fig. 1:**
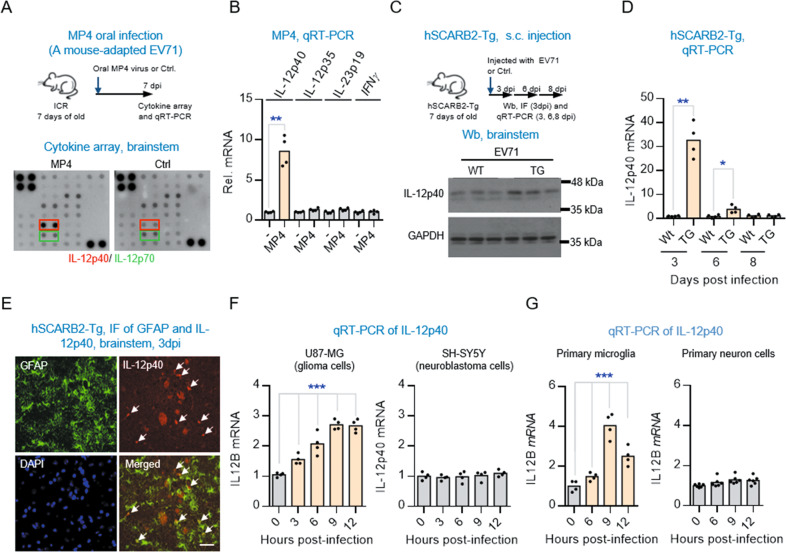
EV71 infection induces an acute IL-12p40 response in the brainstem. **A** Cytokine antibody microarray analysis of brainstem of ICR mice infected with MP4 virus. The ICR mice at 7-day of age were orally infected with or without a 50% lethal dose of a mouse-adapted EV71 strain (MP4) virus and the brainstem tissues were harvested for the analysis at 7 days post-infection (dpi). **B** qRT-PCR analysis of IL-12p40, IL12A, IL23, and IFNγ mRNA levels at 7 dpi in the brainstem of ICR mice infected with or without MP4 virus. Values are represented as mean (*n* = 4). Significantly different from control group: **P < 0.01 by unpaired *t* test. **C** Western blot analysis of IL-12p40 protein levels in the brainstem of hSCARB2-Tg mice infected with EV71 (3 × 10^4^ PFU/mouse). The hSCARB2-Tg (TG) and non-Tg (WT) mice at 7-day of age were injected s.c. with 3 × 10^4^ pfu of 5746 (C2) strain of EV71. The brainstem tissues (*n* = 3) were harvested at the indicated dpi for western blot analysis. D, Temporal expression of IL-12p40 in the brainstem of hSCARB2-Tg (TG) and non-TG (WT) mice infected with EV71. Time-course qRT-PCR analysis of IL-12p40 at the indicated dpi. Values are represented as mean (*n* = 4). Significantly different from control group: *P < 0.05, **P < 0.01 by unpaired *t* test. **E** Representative photomicrographs of anti-GFAP/IL-12p40 immunostaining in the brainstem regions. Scale bars, 50 μm. Time-course qRT-PCR analysis of IL-12p40 mRNA levels in glial and neuronal cells. U87-MG or SH-SY5Y cells were infected with or without EV71 (MOI = 1) and harvested at the indicted hpi for the analysis (**F**). Primary microglia and neurons isolated from hSCARB2-Tg mice were infected with or without EV71 (MOI = 3) and assayed at the indicted hours post-infection (**G**). Values are represented as mean (*n* = 4–6). ***P < 0.001 by One-Way ANOVA.

To begin our investigation into the possible relationship between EV71 and local cellular immune response in the brainstem, we first used a cytokine antibody microarray to profile cytokines and chemokines in the brainstem of mice subjected to a 50% lethal dose of MP4 EV71 (a mouse-adapted EV71 virus) [13] administered by oral infection (Fig. 1A, B, and Fig. S1). IL-12p40 was found to be the only cytokine prominently upregulated seven days post-infection (dpi). This finding was confirmed by qRT-PCR analysis (Fig. 1B). To determine whether brainstem was a major brain component with IL-12p40 response to EV71 infection, we also examined the IL-12p40 protein and mRNA levels in cerebrum by using cytokine microarray analysis. Interestingly, the IL-12p40 protein levels showed no notable change in cerebral tissue aftter oral infection of EV71 in the mice, suggesting that the EV71-induced IL-12p40 response in brainstem was a local effect (Fig. S1A and B).

IL-12 is a heterodimeric cytokine encoded by two separate genes, IL-12A (p35) and IL-12B (p40) [18]. It has been shown that p40 to be secreted as a monomer and homodimer in the mediation of distinct autoimmune signaling pathways [19]. Interestingly, we found no evidence for early induction of other members of the IL-12 family, including IL-12p35, IL-12p70, or IL-23p19 in the brainstem of EV71-infected mice (Fig. 1B). To confirm our findings, we performed another experiment using EV71-infected hSCARB2-Tg mice, which are transgenic mice of human EV71 receptor (scavenger receptor B2) to enable infection [17]. Elevated IL-12p40 levels were also found in the brainstems of these mice (Fig. 1C, D, and Fig. S2). Together, these results suggest EV71 induction of the cytokine IL-12p40 may be involved in the pathogenesis of the brainstem encephalitis during severe EV71 infection.

We next studied the temporal expression pattern of IL-12p40 at different time points post infection. Most notably, the brainstem tissue of the mice inoculated with EV71 virus showed an acute and transient upregulation of IL-12p40 at the early infection phase and declined sharply (Figs. 1D and S2D). Additionally, we also measured the IL-12p40 levels in serum and found they also showed a similar dynamic expression pattern (Fig. S3).

Because antibody to EV71 has been detected in both the neurons and astrocytes in the brain tissues of the EV71-infected human patients [20], we were interested in knowing whether IL-12p40 was selectively expressed in glial and/or neuronal cells in the brainstem of EV71-infected hSCARB2-Tg mice (Fig. 1E). In an immunohistochemical study counterstaining IL-12p40 with GFAP, we found notably localized positive staining signals of IL-12p40 in loci of astroglia cells, suggesting that the EV71-induced IL-12p40 expression was distributed predominantly in the astroglia cells. We also used the U87-MG glioma cells, SH-SY5Y neuroblastoma, primary microglia and neurons isolated from hSCARB2-Tg mice for the EV71 infection to determine if there was a cell-type difference in expressing IL-12p40. As seen in our qRT-PCR analysis, IL-12p40 expression was only induced in microglial cells, not in neuronal cells (Fig. 1F, G and Fig. S4A). Western blot analysis further confirmed that IL-12p40 response was only induced in glial cells (Fig. S5). To test whether this was a serotype-specific host immune response pattern in brainstem, we also used coxsackievirus A16 (CA16), another common but less neurovirulent cause of HFMD, to perform the same experiment. CA16 induced a similar but less pronounced IL-12p40 response in U87-MG cells (Fig. S4B). These results suggest that the EV71 infection may induce an early strong IL-12p40 response in the brainstem.

### IL-12p40’s response to EV71 leads the development of encephalitis

**Fig. 2:**
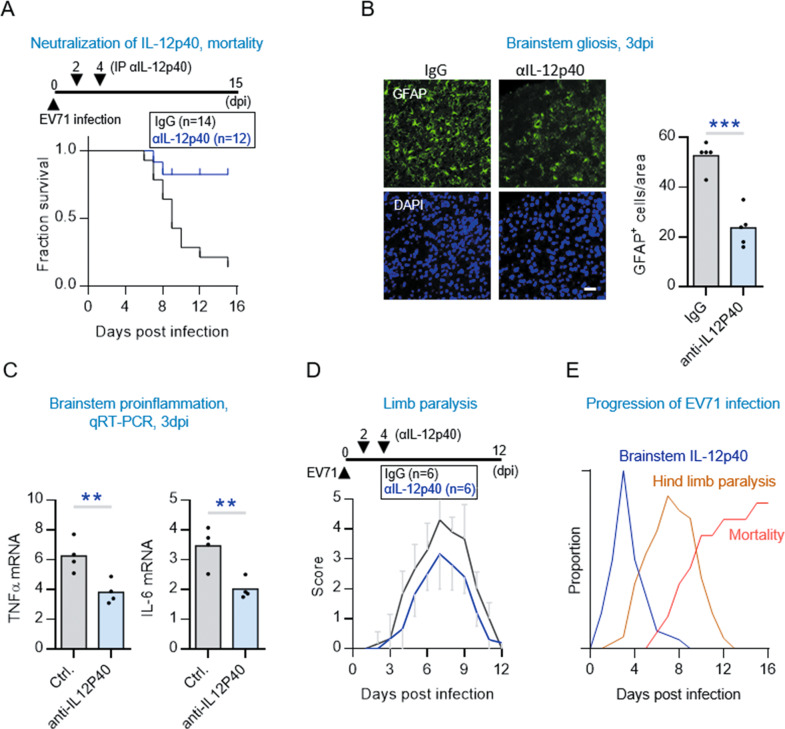
The IL-12p40 response is essential in the pathogenesis of EV71-induced brainstem encephalitis. **A** Kaplan-Meier survival curve of EV71-infected hSCARB2-Tg mice receiving treatment with anti-IL-12p40 antibody or control IgG. The 7-day-old hSCARB2-Tg mice were injected s.c. with 3 × 10^4^ pfu of EV71 and subsequently intraperitoneally injected with anti-IL12p40 antibody (2 ug/mouse) or IgG (Ctrl) at 2 and 4 dpi. Significantly different from control group at *p* < 0.01 by Log Rank test. **B** Representative photomicrographs and quantification of anti-GFAP immunostaining in the brainstem regions of the EV71-infected hSCARB2-Tg mice treated with anti-IL-12p40 antibody or control IgG. Scale bars, 50 μm. The GFAP-positive signals in five consecutive sections per animal was quantified by ImageJ and presented as mean (*n* = 5, right panel). Significantly different from control group: ***P < 0.01 by unpaired *t* test. **C** qRT-PCR analysis of TNFa and IL6 mRNA levels in the brainstem of the EV71-infected hSCARB2-Tg mice treated with anti-IL-12p40 antibody or control IgG. Values are represented as mean (*n* = 4). Significantly different from control group at *p < 0.05 by unpaired *t* test. **D** Progressive limb paralysis in the EV71-infected hSCARB2-Tg mice treated with anti-IL-12p40 antibody or control IgG. Limb paralysis (LP) is defined as the rigidness of mouse legs which are hesitate to move. For scoring LP; 5 = severe front and rear LP and no movement, 4 = moderate two rear LP and hesitant movement, 3 = one rear LP with bending legs, 2 = mild rear limb bended, 1 = slightly rear limb bended, 0 = normal movement. Significantly different from control group: *p* < 0.001 by unpaired *t* test. **E** The configuration of temporal sequential order of the occurrence of IL-12p40 expression, limb paralysis, and mortality in the EV71-infected hSCARB2-Tg mice.

After discovering that IL-12p40 was the only prominent cytokine induced during the early EV71 infection stage, we wanted to study what impact this response might have on the pathogenesis of brainstem encephalitis. To do this, we treated infected mice with monoclonal anti-IL-12p40 antibody to neutralize the effect of IL-12p40. Significantly, the injection of antibody resulted in a marked reduction in mortality in the infected mice compared to controls (Fig. 2A). This result is important because it showed that the early induction of IL-12p40 signaling was a key detrimental host response module associated with fatal outcome of EV71 infection.

Since fatal outcome is most commonly associated with brainstem encephalitis, we next investigated whether the IL-12p40 response played a major role in development of encephalitis. To find out, we accessed three main characteristics features of brainstem encephalitis, which were proinflammatory cytokine level, brainstem gliosis, and limb paralysis, in the EV71-infected mice untreated and treated with anti-IL-12p40 antibody (Fig. 2B–D). In an immunohistologic study, we stained the glial fibrillary acidic protein (GFAP) in brainstem sections obtained from infected mice treated and untreated with the antibody, and found a significant reduction in GFAP signals in those treated with the antibody, compared to the untreated mice (Fig. 2B). Because EV71 patients with encephalitis have been reported to have increased TNFα and IL-6 levels [21], we also investigated whether the gliosis-associated inflammatory markers would also be decreased in sera collected from these mice treated with the antibody. TNFα and IL-6 levels were indeed found to be significantly decreased in the treated mice compared to the untreated controls (Fig. 2C). Moreover, since limb paralysis is a typical neurological sign of brainstem damage caused by EV71 in mouse models [22], we assessed limb paralysis in our infected mice over time post infection [17]. Mice treated with the antibody showed moderate improvement in limb paralysis in general (Fig. 2D). Considered together, these results indicate that the anti-IL-12p40 antibody treatment resulted in a great reduction in the three characteristic features of brainstem encephalitis, suggesting that the EV71-induced IL-12p40 response is key to the development of brainstem encephalitis.

During the course of our experiments, we noticed that onset and worsening of limb paralysis appeared to follow a temporal pattern correlating with the expression pattern of IL-12p40. Therefore, we decided to plot the temporal dynamics of the changes in the IL-12p40 expressions, limb paralysis, and mortality over time post infection. In brief, the onset of limb paralysis was correlated with the peak of IL-12p40 levels and the onset of mortality was correlated with peak limb paralysis (Fig. 2E). Therefore, in response to infection, the dynamics surrounding IL-12p40 expression, limb paralysis, and mortality are functionally linked in the mouse brainstem.

### TLR9 mediates the EV71-induced IL-12p40 response

**Fig. 3:**
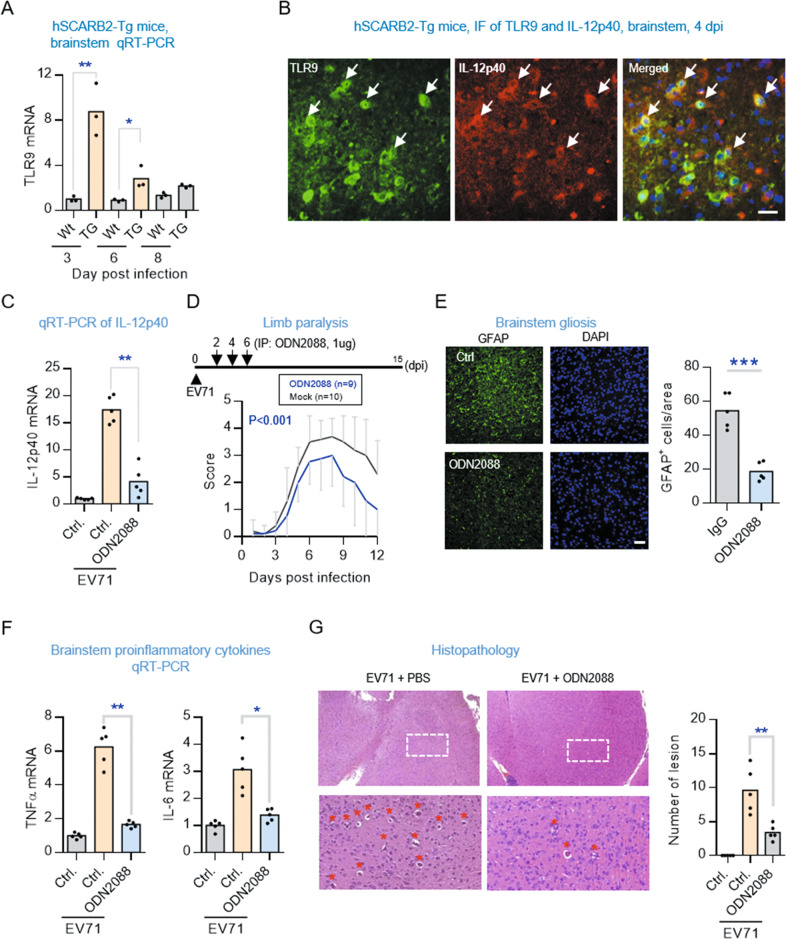
TLR9 is required for EV71 to elicit an IL-12p40 response and include brainstem encephalitis. **A** Temporal expression pattern of TLR9 in the brainstem of hSCARB2-Tg (TG) and non-TG (WT) mice infected with EV71. qRT-PCR analysis of TLR9 at the indicated dpi. Values are represented as mean (*n* = 3). Significantly different from control group: *P < 0.05, **P < 0.01 by unpaired *t* test. **B** Representative photomicrographs of TLR9 (green) colocalized with IL-12p40 (red) in the brainstem regions of mice infected with EV71 at 4dpi. Scale bars, 40 μm. **C** qRT-PCR analysis of IL-12p40 expression levels in the brainstem of the EV71-infected hSCARB2-Tg mice with the treatment ODN2088 or control IgG. Values are represented as mean (*n* = 5). Significantly different from control group: **P < 0.01 by unpaired *t* test. **D** EV71-induced limb paralysis at indicated dpi in hSCARB2-Tg mice treated with or without ODN2088. Significantly different from control group: *p* < 0.001 by unpaired *t* test. **E** Representative photomicrographs of anti-GFAP immunostaining in the brainstem regions of the EV71-infected hSCARB2-Tg mice treated with or without ODN2088 at 3 dpi. Scale bars, 50 μm. The GFAP-positive signals in five consecutive sections per animal (*n* = 5) was quantified by ImageJ and presented as mean ± SD (right panel). Significantly different from control group: ***P < 0.001 by two-tailed unpaired *t* test. **F** qRT-PCR analysis of TNFα and IL-6 mRNA levels in the brainstem of the EV71-infected hSCARB2-Tg mice treated with or without ODN2088. Values are represented as mean (*n* = 5). Significantly different from control group: *p < 0.05 by two-tailed unpaired *t* test. **G** Histopathological examination of brainstem in the EV71-infected hSCARB2-Tg mice treated with ODN2088 or ctrl. Asterisks (∗) on the field of pathological foci with monocytes/macrophages infiltration. Number of lesion in five consecutive sections per animal (*n* = 5) was quantified and presented as the mean. Significantly different from control group, **P < 0.01 by unpaired *t* test.

EV71 is known to enter host cells by binding with cell surface receptor SCARB2 or PSGL-1 mediating endocytotic delivery of the viral genome into the cytosol [23]. The viral genome into the cytoplasm is subsequently detected by the host’s innate immune system via intracellular receptors. We became interested in what intracellular sensor might initiate the IL-12p40 response to EV71 infection. The virus genome of EV71 is a non-enveloped, positive single-stranded RNA (ssRNA). Toll-like receptors (TLRs) are key pattern recognition receptors (PRRs) able to detect both cell surface and intracellular conserved molecular signatures of microbial pathogens. Of the TLR family, four members (TLR3, TLR7, TLR8, and TLR9) are known intracellular sensors able to detect distinct forms of viral nucleic acids in the endosome. TLR7 and TLR8 are critical to recognizing single-stranded RNA (ssRNA), while TLR3 and TLR9 recognize viral double-stranded RNA and unmethylated CpG DNA, respectively [24]. However, one animal study recently discovered that TLR7 is not the intracellular PRR used to recognize ssRNA of EV71 in mouse spinal cord [25]. Because the expression of many TLR genes changes when these receptors are engaged with microbial ligands to initiate immune responses [26–28], we examined which of these four intracellular TLRs would be particularly upregulated in the brainstem of hSCARB2-Tg mice after infection with EV71. The qRT-PCR results showed a marked increase in TLR9, a moderate increase in TLR3 and TLR7, and less obvious increase in TLR8 mRNA levels (Fig. 3A, B and Fig. S6A).

We considered TLR9 might be a potential intracellular sensor in mediating the EV71-induced IL-12p40 expression, bescuse TLR9 can activate IRF5 transcription activity involved in the upregulation of IL-12p40, while TLR3 selectively activates IRF3 for IL-12p35 (Fig S6B) [29, 30]. To test this hypothesis, we administered an inhibitory CpG oligodeoxynucleotide (CpG ODN) to infected mice to block TLR9 activation. We found a significant reduction in IL-12p40 response, limb paralysis, gliosis, proinflammatory cytokines, and histopathological lesion in the brainstem of infected mice treated CpG ODN, compared to untreated infected controls (Fig. 3C–G and Fig. S7). These results support the hypothesis that TLR9 mediates the induction of IL-12p40 response and subsequent development of brainstem encephalitis in mice infected with EV71.

### IL-12p40 elicits neurotoxic effect through iNOS/NO signaling

**Fig. 4:**
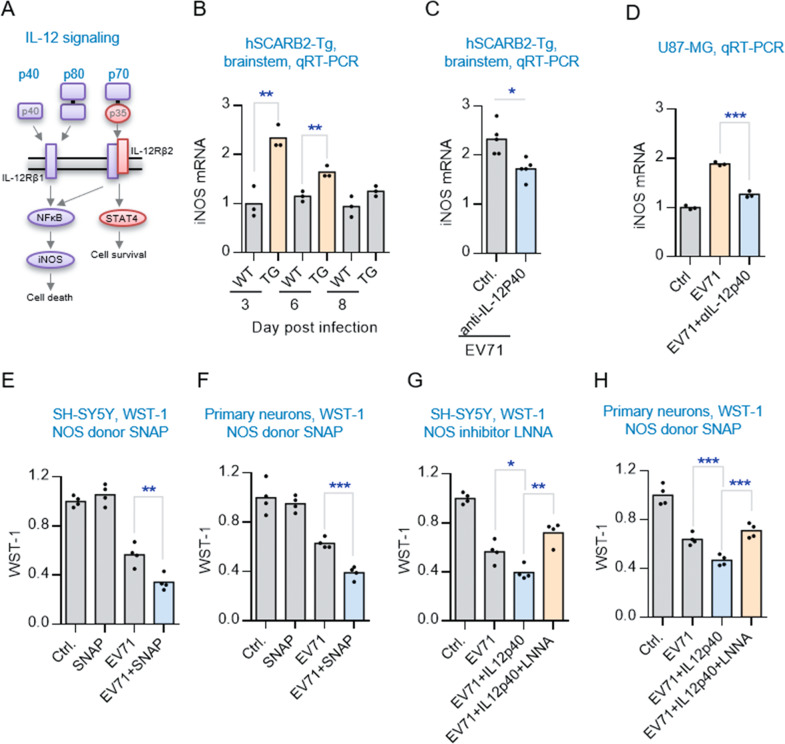
IL-12p40 promotes neuronal damage through activating iNOS/NO response. **A** Graphical illustration of how different subunits of IL-12 interact with specific cell surface receptors in controlling distinct downstream transcription factors and signaling events. **B** qRT-PCR analysis of iNOS expression levels in the brainstem of hSCARB2-Tg (TG) or non-TG (WT) mice during the time course of EV71 infection. Values are represented as mean (*n* = 3). Significantly different from control group: **P < 0.01 by unpaired *t* test. **C** qRT-PCR analysis of iNOS expression levels in the EV71-infected hSCARB2-Tg mice treated with or without anti-IL-12p40 antibody (*n* = 5). **D** qRT-PCR analysis of iNOS expression levels in the EV71-infected U87-MG cells treated with or without anti-IL-12p40 antibody (*n* = 3). Effect of NO level on EV71-induced cell viability. SH-SY5Y and primary neuronal cells were pre-treated with or without NO donor SNAP (**E**, **F**) or Nitric oxide synthase inhibitor LNNA (**G**, **H**) 1 h before infection with EV71 (MOI = 1 for SH-SY5Y and MOI = 3 for primary neuron). WST-1 analysis was conducted at 9 hpi. Values are represented as mean (*n* = 4). Significantly different from control group at *p < 0.05, **p < 0.01 by unpaired *t* test.

Because IL-12p40 has been previously shown to induce the expression of nitric oxide synthase (iNOS) in glial cells in the modulation of neuroinflammatory diseases (Fig. 4A) [31, 32], we tested whether IL-12p40 affected iNOS levels in the brainstem of virus-infected hSCARB2-Tg mice. Indeed, qRT-PCR results showed that the iNOS levels in the brainstems of these mice were significantly increased. Importantly, treatment of neutralizing antibody against IL-12p40 resulted in the reduction of iNOS levels (Fig. 4B, C), a finding suggesting that IL-12p40 was indeed involved in the modulation of iNOS expression in the brainstem. To exclude the possibility of an indirect IL-12p40 effect on iNOS from non-glial cells, we conducted the same experiment using a U87-MG glial cell line and found that EV71-induced iNOS expression was significantly reduced by anti- IL-12p40 antibody (Fig. 4D), further supporting the role of IL-12p40 in inducing iNOS expression in the EV71-infected glial cells.

To further investigate the potential involvement of iNOS on the EV71-induced neuronal apoptosis, we used a NO donor SNAP to chemically increase cellular NO level. Indeed, the SNAP treatment significantly increased EV71-induced neuronal apoptosis (Fig. 4E, F). Oppositely, suppression of NO by a NOS inhibitor L-NNA markedly decreased the EV71-induced neuronal apoptosis (Fig. 4G, H). Thus, these results suggest that the induction of iNOS/NO-signaling pathway by IL-12p40 is responsible for the EV71-induced neurotoxicity.

## Discussion

Although host production of cytokines can limit the spread of viral infections, this response can also become pathological when it is excessively activated. This study found that EV71 engagement with intracellular TLR9 elicited a neurotoxic glial response through IL12p40-iNOS signaling leading to the neurological manifestations of EV71 infection, which in this study was brainstem encephalitis. COVID-19 is the recent best-known disease that caused by an excessive inflammatory response in promoting respiratory failure in the infected patients [33]. Intriguingly, the typical respiratory symptoms caused by COVID-19 infection are often associated with neurological symptoms in severe cases and accompanied by elevated serum IL-12 levels [34–38]. Because our study has demonstrated that the EV71-induced neurological complications may be mediated by the TLR9 induction of IL-12p40 response in the brainstem, there may be some TLR9 involvement in the development of manifestations of COVID-19 infection. In fact, it has recently been proposed that TLR9 plays a role in the development of severe COVID-19 manifestations and that TLR9 be targeted therapeutically [39]. The most attractive feature of this model is that several TLR9-blocking drugs have already entered for other purposes clinical trials and could be fast-tracked for development if urgently needed.

Previously, TLR9 was only known as an intracellular receptor that recognizes bacterial unmethylated DNA [40]. However, our results clearly show that TLR9’s sensing of an EV71 infection is needed to elicit a hyper IL-12p40 response. Similarly, the finding of one previous study suggests TLR9 may also function as a sensor of another ssRNA virus, dengue [41]. That study and ours have identified a new role of TLR9, one as an intracellular sensor for viral ssRNA in the innate immune system.

Since TLR9 can act as an intracellular sensor for viral ssRNA and thereby induce IL-12p40 response, it would be relevant to find whether other ssRNA viruses might trigger IL-12p40 responses. In fact, infections by parechovirus, an ssRNA virus, have already been shown to elevate the plasma IL-12p40 in young children [42], though it is not known whether this response is also mediated through TLR9. Respiratory influenza A virus, also an ssRNA virus, has been reported to induce a host IL-12p40 response mediated by TLR7 [43, 44]. Based these findings and ours, it is likely that the ssRNA viruses might have evolved different strategies for using the various intracellular TLRs to manipulate host cytokine responses.

Given that this study shows IL-12p40 response is critical for the development of EV71-induced neurological complications, it would be of interest to investigate whether IL-12p40 fulfills this function alone or it does so by forming a heterocomplex with other IL-12 family members. The p40 subunit commonly forms partners with the p35 and p19 to generate the IL-12p70 and IL-23, respectively. However, our cytokine antibody microarray and qPCR experiments did not identify IL-12p70 or IL-23 protein levels to be differentially regulated in the brainstem of mice exposed to EV71 infection. Because the p40 subunit also exists extracellularly as either a monomer or homodimer, we suspect that either a monomer or homodimer of IL-12p40 may be involved in the development of neurological complications. Previous studies have suggested that both the monomer or homodimer of IL-12p40 might induce an increase in iNOS expression by stimulating IL-12Rβ1-mediated activation of NF-κB [31, 45]. Our study has found iNOS signaling involvement in IL-12p40 effect on neurotoxicity, suggesting that both monomer and homodimer forms of IL-12p40 could behave synergistically or complementarily to cause adverse immune effects in brainstem encephalitis.

The pivotal function of the immune system when faced with a microbial infection is to maintain the homeostasis between the pathogen control and tolerance within the individual to sustain host integrity. While immunodeficiency may play one role in the earlier onset manifestations of neurological damage, excessive immune activity can also lead to tissue damage by the release of toxic substances exemplified by pathological inflammation observed in patients with EV71 infection. It should be noted that although our study showed that blocking the TLR9-mediated IL-12p40 response ameliorated brainstem encephalitis caused by severe EV71 infection in a mouse model, it is likely that the IL-12p40 response plays a dual role during the infection. Therefore, future studies might want to investigate optimal timing and duration of treatment when targeting IL-12p40 response in order to control both the hyper- and hypo-inflammatory phases.

**Fig. 5:**
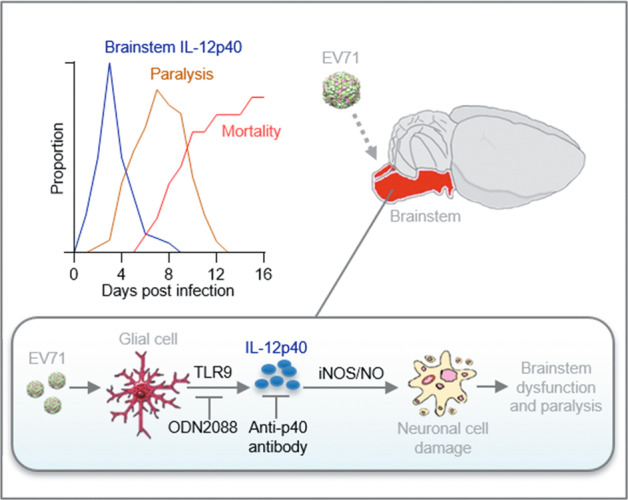
Graphical summary of major findings of this study on the TLR9-IL-12p40-iNOS signaling pathway in the EV71-induced brainstem encephalitis. Severe EV71 infection induces an acute IL-12p40 response through intracellular sensor TLR9 in glia cells of the brainstem, subsequently generating neurotoxic NO causing encephalitis. These findings provide support for the therapeutic value of blocking the TLR9 mediated IL-12p40 response in the treatment of severe EV71 infection-induced brainstem encephalitis.

In conclusion, as can be seen in the graphical model in Fig. 5, TLR9 sensing of EV71 increases the expression of IL12-40 in the glia where neurotoxic iNOS/NO signaling is induced causing damage to the brainstem.

## Supplementary information

Supplementary Figure legends

Supplemental Fig. S1

Supplemental Fig. S2

Supplemental Fig. S3

Supplemental Fig. S4

Supplemental Fig. S5

Supplemental Fig. S6

Supplemental Fig. S7

Original Data File (Wb and IF image)

Original Data File (Value data)

Checklit

## Data Availability

The data that support the findings of this study are available from the corresponding author upon reasonable request and with permission of NHRI.
