# Supplementary material for: Neurotropic EV71 causes encephalitis by engaging intracellular TLR9 to elicit neurotoxic IL12-p40-iNOS signaling
Source: Cell Death Dis. 2022 Apr 11;13(4):328. doi: 10.1038/s41419-022-04771-3 (PMC8995170; doi:10.1038/s41419-022-04771-3)
Supplement: Supplementary file 1 — Supplementary Figure legends [file 41419_2022_4771_MOESM1_ESM.docx]

**Supplementary Figure legends**

Fig S1. IL-12p40 response in cerebral tissues of mice treated with oral or subcutaneous injection of EV71. (A) Cytokine array analysis of IL-12p40 response in the cerebrum of ICR mice oral infected with EV71/MP4 virus. (B) Graphical illustration of experimental setup and cytokine microarray analysis showing cerebrum increased IL-12p40 level after i.p injection of MP4 virus. (C) Panel of cytokine array in this study. The panel including 22 targets: G-CSF, GM-CSF, IL-2, IL-3, IL-4, IL-5, IL-6, IL-9, IL-10, IL-12 p40, IL-12 p70, IL-13, IL-17, IFNg, MCP-1, MCP-5, RANTES, SCF, sTNFRI, TNF-a, Thrombopoietin and VEGF.

Fig S2. Temporal levels of IL-12p35 and IL-23p19 transcripts as well as viral burden during the time course of infection, and gliosis at 3 dpi in the brainstem of EV71-infected hSCARBS-Tg mice. (A), qRT-PCR analysis of IL-12p35 and IL-12p19 in the brainstem of EV71-infected hSCARB2-Tg (TG) or non-Tg (WT) mice during the time course of infection. Values of qRT-PCR are represented as mean (n=3). (B), Viral burdens in the brainstem of EV71-infected hSCARB2-Tg mice at the indicated dpi (n=3). (C), Representative immunofluorescence image of anti-GFAP staining at 3 dpi in the brainstem of 7-d-old hSCARB2-Tg (TG) or non-TG (WT) mice s.c. injected with 3×10^4^ pfu of 5746 (C2) strain of EV71. Scale bars, 40 μm. (D), qRT-PCR analysis of IL-12p40 in the brainstem of EV71-infected (EV71) or non-infected (Ctrl) hSCARB2-Tg mice during the time course of infection. Values of qRT-PCR are represented as mean (=3). Significantly different from control group at *P<0.05 or ***p<0.001 by unpaired t-test.

Fig S3. Temporal levels of serum IL-12p40 levels in EV71-infected hSCARBS-Tg mice during the time course of infection. Sera were collected from two independent experiments for ELISA assays (n= 3-5 mice per each experiment). Values are represented as the mean ± SD. Significantly different from control group at *p <0.05, ***P<0.001 by unpaired t-test.

Fig S4. Temporal levels of IL-12p35 and IL-p19 transcripts as well as viral burdens in U87-MG cells infected by EV71 (A) and IL-12p40 levels by CA16 (B) during the time course of infection.

Fig S5. Western blot analysis of IL-12p40 levels in the EV71-infected U87-MG and SH-SY5Y cells. The secreted IL-12p40 levels in the culture medium (250 μL) were harvested for analysis at the indicated hpi.

Fig S6. Transcript levels of three different TLR family members in the brainstem tissues and graphical illustration showing two TLR-dependent pathways in mediating expressions of IL-12 family members. (A) Temporal levels of TLR3, TLR7, and TLR8 transcripts during the time course of infection in the brainstem of EV71-infected hSCARB2-Tg (TG) or non-TG (WT) mice. qRT-PCR values are represented as mean (n=4). Significant difference from control group set at *p<0.05, **p<0.01 and ***p<0.001 by unpaired t-test. (B) Graphical illustration showing distinct signaling pathways are engaged by TLR3 and TLR9 in mediating the induction of IL-12p35 and IL-23p40 expressions, respectively.

Fig S7. Graphical illustration of experimental setup designed for the treatment of the EV71-infected hSCARBS-Tg mice with TLR9 antagonist ODN2088.
