# Supplementary figures and images for: Neurotropic EV71 causes encephalitis by engaging intracellular TLR9 to elicit neurotoxic IL12-p40-iNOS signaling
Source: Cell Death Dis. 2022 Apr 11;13(4):328. doi: 10.1038/s41419-022-04771-3 (PMC8995170; doi:10.1038/s41419-022-04771-3)

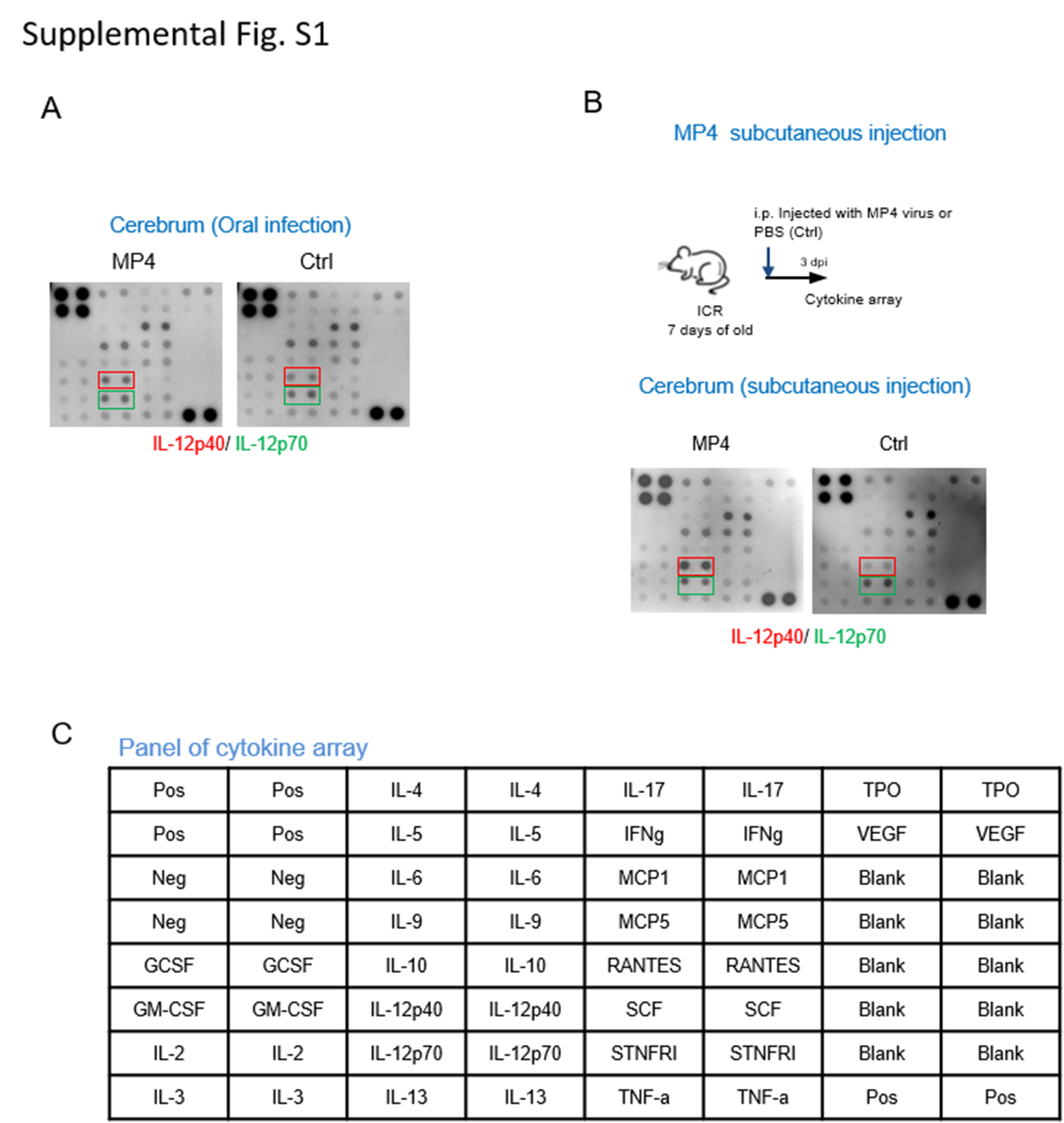

Supplement: Supplementary file 2 — Supplemental Fig. S1 [file 41419_2022_4771_MOESM2_ESM.tif]

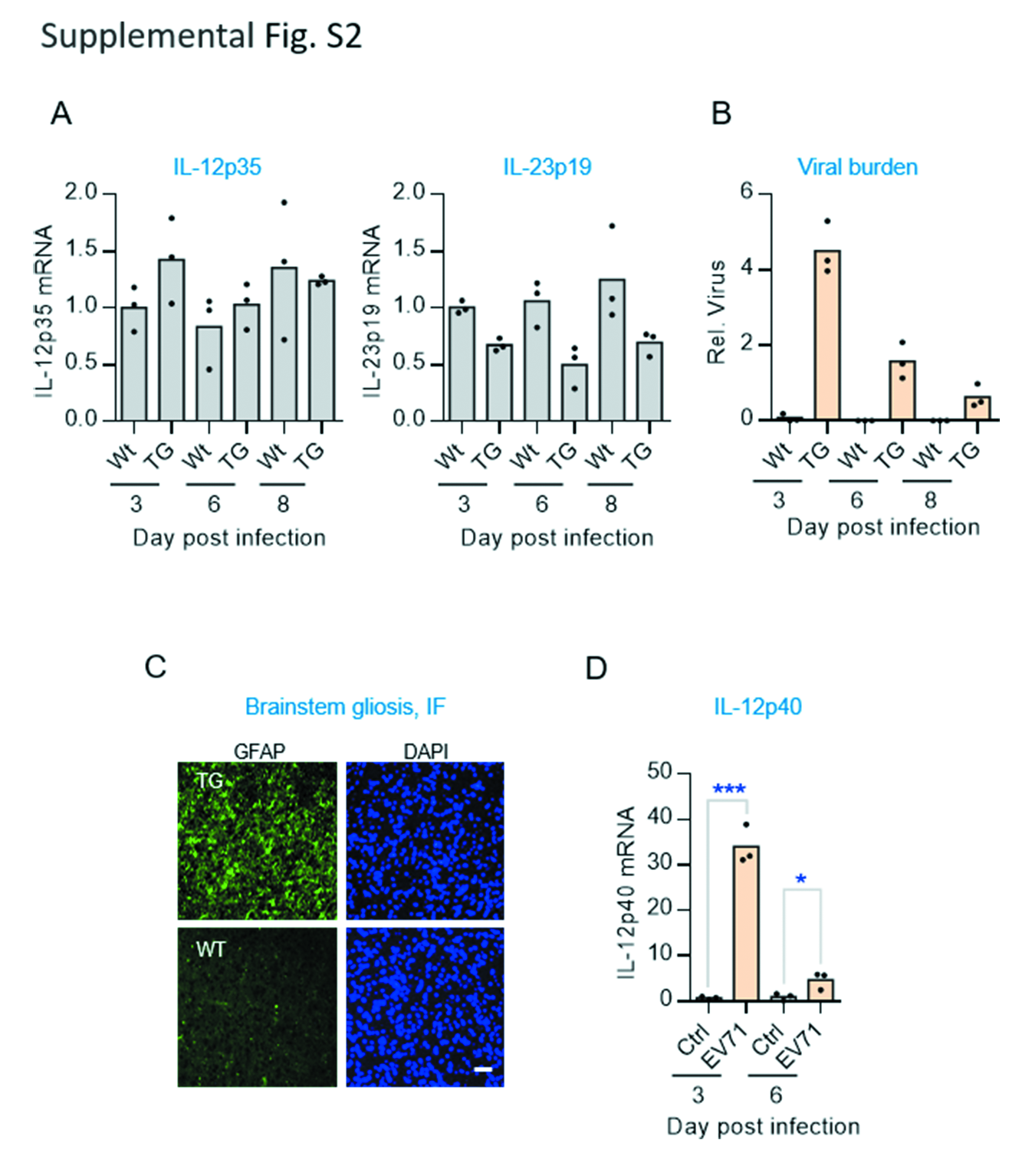

Supplement: Supplementary file 3 — Supplemental Fig. S2 [file 41419_2022_4771_MOESM3_ESM.tif]

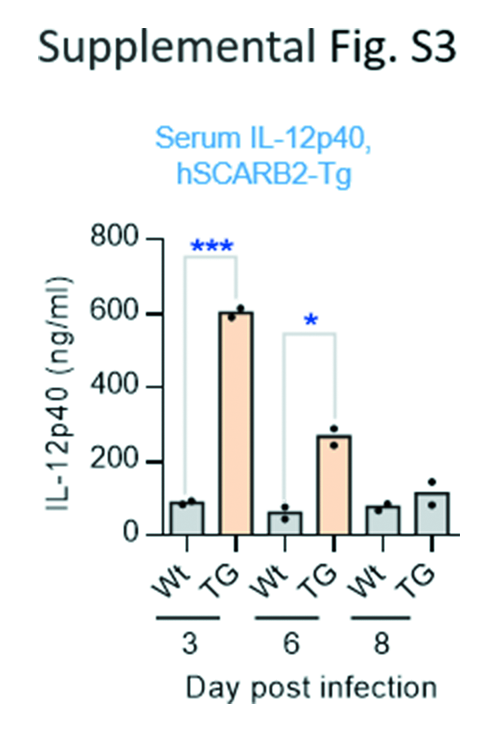

Supplement: Supplementary file 4 — Supplemental Fig. S3 [file 41419_2022_4771_MOESM4_ESM.tif]

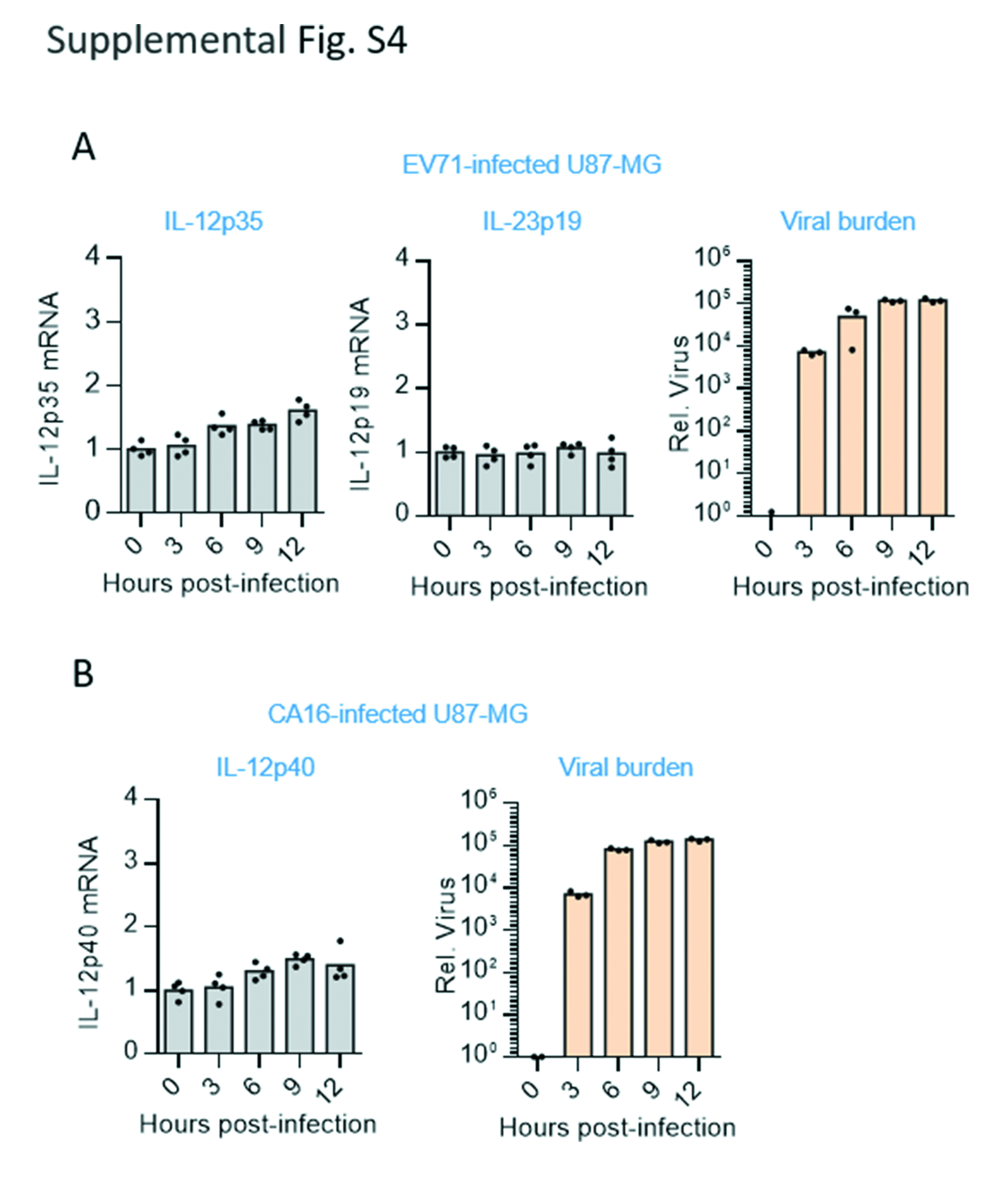

Supplement: Supplementary file 5 — Supplemental Fig. S4 [file 41419_2022_4771_MOESM5_ESM.tif]

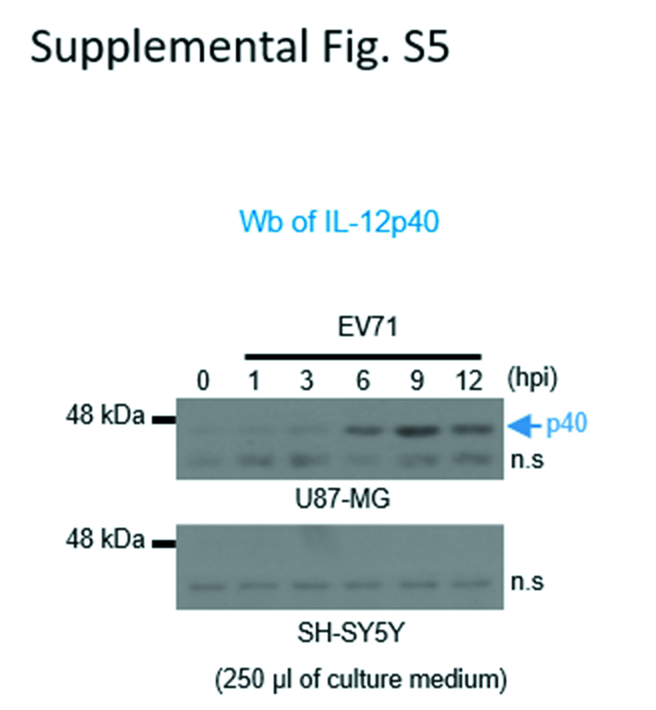

Supplement: Supplementary file 6 — Supplemental Fig. S5 [file 41419_2022_4771_MOESM6_ESM.tif]

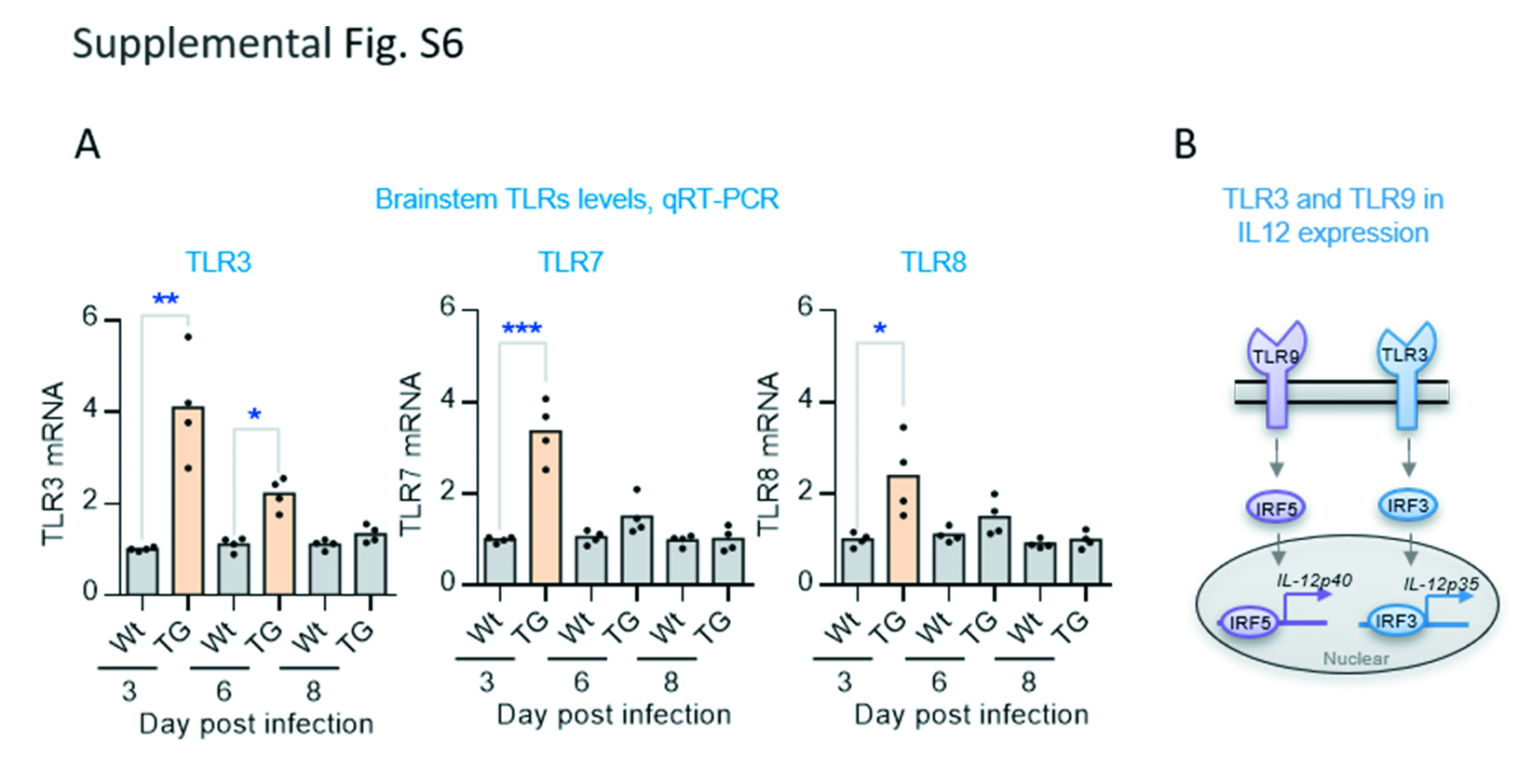

Supplement: Supplementary file 7 — Supplemental Fig. S6 [file 41419_2022_4771_MOESM7_ESM.tif]

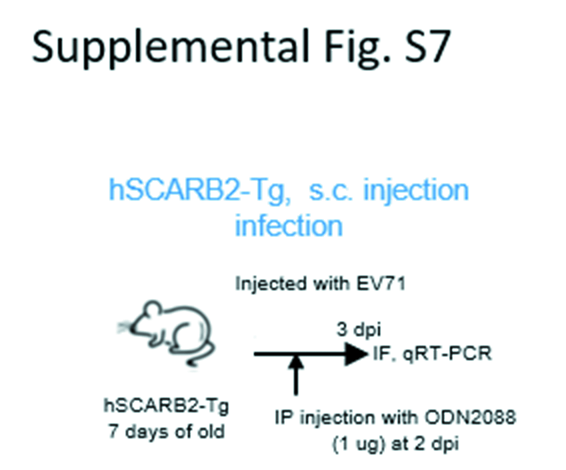

Supplement: Supplementary file 8 — Supplemental Fig. S7 [file 41419_2022_4771_MOESM8_ESM.tif]
