# Supplementary material for: Neurotropic EV71 causes encephalitis by engaging intracellular TLR9 to elicit neurotoxic IL12-p40-iNOS signaling
Source: Cell Death Dis. 2022 Apr 11;13(4):328. doi: 10.1038/s41419-022-04771-3 (PMC8995170; doi:10.1038/s41419-022-04771-3)
Supplement: Supplementary file 9 — Original Data File (Wb and IF image) [file 41419_2022_4771_MOESM9_ESM.pptx]

## Slide 1
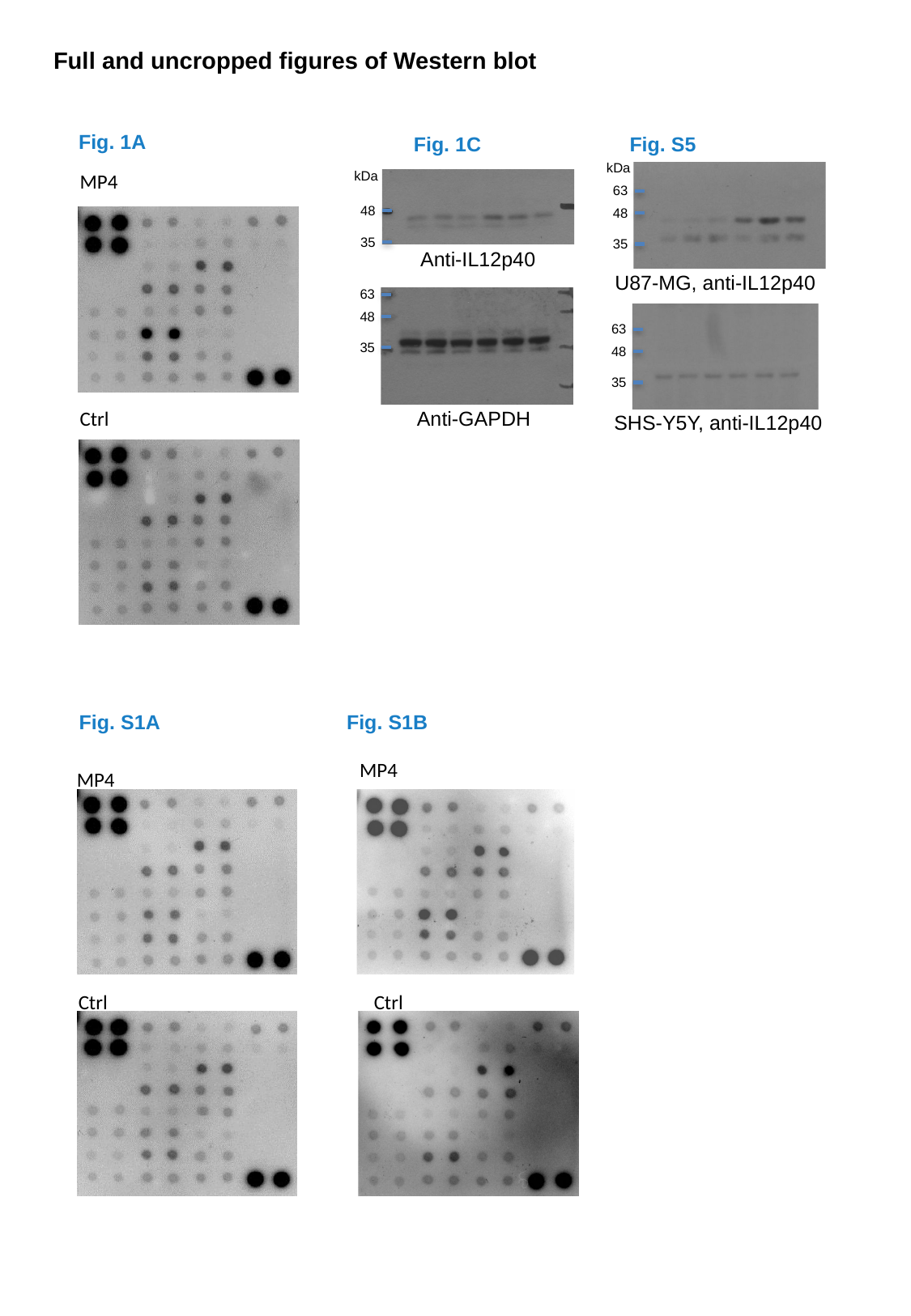

Full and uncropped figures of Western blot
Fig. 1A
Fig. 1C
Fig. S5
kDa
kDa
MP4
63
48
48
35
35
Anti-IL12p40
U87-MG, anti-IL12p40
63
48
63
35
48
35
Ctrl
Anti-GAPDH
SHS-Y5Y, anti-IL12p40
Fig. S1A
Fig. S1B
MP4
MP4
Ctrl
Ctrl

## Slide 2
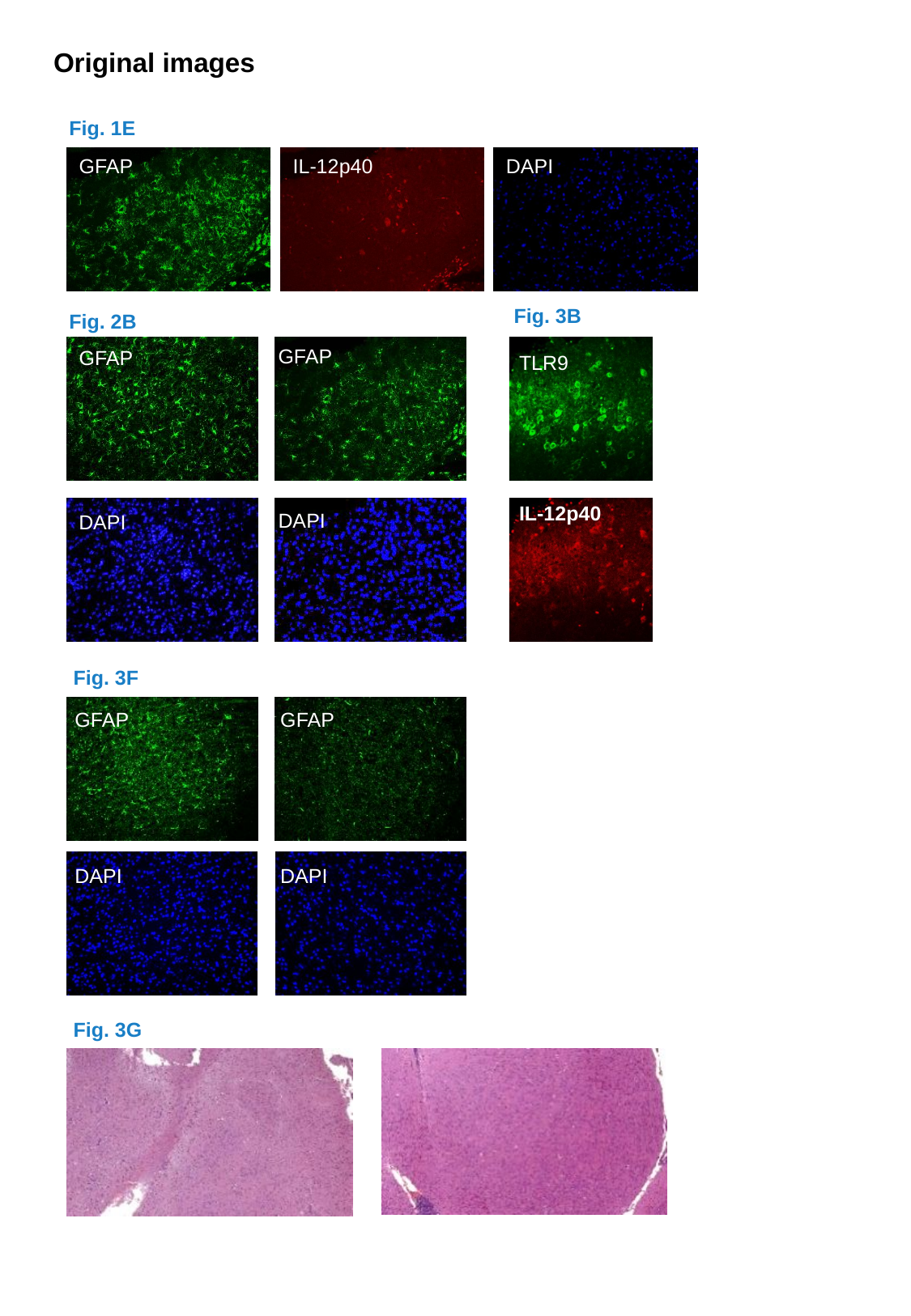

Original images
Fig. 1E
GFAP
IL-12p40
DAPI
Fig. 3B
Fig. 2B
GFAP
GFAP
TLR9
IL-12p40
DAPI
DAPI
Fig. 3F
GFAP
GFAP
DAPI
DAPI
Fig. 3G
